# Maternal blood lead levels and proximity to legacy mining contamination in Kabwe, Zambia: socioeconomic disparities and potential implications on fetal health

**DOI:** 10.1007/s11356-026-37774-0

**Published:** 2026-04-28

**Authors:** Doreen Sakala, John Yabe, Isaac Fwemba, Nosiku Munyinda, Nasson Nathan Tembo, Madalitso Tembo, Hikabasa Halwiindi, Mayumi Ishizuka, Shouta M. M. Nakayama

**Affiliations:** 1 Department of Public Health, Ministry of Health, P.O. Box 30205, Lusaka, Zambia; 2 Department of Para‑Clinical Studies, School of Veterinary Medicine, University of Namibia, P/B 13101 Windhoek, Namibia; 3 Department of Medical Education, School of Medicine, University of Zambia, P.O. Box 32379, Lusaka, Zambia; 4 Department of Environmental Health, School of Public Health, University of Zambia, P.O. Box 32379, Lusaka, Zambia; 5 Department of Public Health, School of Medicine and Health Sciences, Mulungushi University, P.O. Box 60009, Livingstone, Zambia; 6 Department of Biomedical Sciences, School of Veterinary Medicine, University of Zambia, P.O. Box 32379, Lusaka, Zambia; 7 Department of Community and Family Medicine, School of Public Health, University of Zambia, P.O. Box 32379, Lusaka, Zambia; 8 Laboratory of Toxicology, School of Veterinary Medicine, Hokkaido University, Kita 18, Nishi 9, Kita-Ku, Sapporo, Japan; 9 Department of Para‑Clinical Studies, School of Veterinary Medicine, University of Zambia, P.O. Box 32379, Lusaka, Zambia; 10 Department of Epidemiology and Biostatistics, School of Public Health, University of Zambia, P.O. Box 32379, Lusaka, Zambia

**Keywords:** Blood lead levels, Maternal exposure, Legacy contamination, Socioeconomic disparities, Fetal health risks, Kabwe, Zambia

## Abstract

Legacy lead (Pb) contamination from a century-long Pb-Zn mining operation (1906–1994) continues to pose severe environmental health threats in Kabwe, Zambia, one of the world’s most polluted sites. While elevated blood lead levels (BLLs) in children are well-documented, maternal exposure remains understudied despite its critical implications for fetal development. We conducted a cross-sectional biomonitoring study among 510 pregnant women across four townships in Kabwe (Makululu, Kasanda, Katondo, and Mahatma Gandhi), stratified by proximity to the former mine (1.5–4.5 km). Sociodemographic and clinical data were collected utilizing a structured questionnaire. Venous blood samples were analyzed for Pb using graphite furnace atomic absorption spectrophotometry (GFAAS), with rigorous quality control (recovery: 92.3–95.1%; MDL: 0.012 µg/dL). Maternal BLLs varied significantly by township (*p* < 0.001): median BLLs were highest in Makululu (6.5 µg/dL; 84.4% elevated), followed by Kasanda (3.4 µg/dL; 48.1%), and lowest in Katondo (1.8 µg/dL; 10.0%) and Mahatma Gandhi (1.6 µg/dL; 8.8%). Overall, 43.7% of women exceeded the reference level of 3.5 µg/dL, disproportionately affecting those in the informal settlements near the mine waste. Elevated BLLs were significantly associated with longer years of stay in the area (*p* = 0.007), unemployment (*p* < 0.001), smoking (*p* = 0.021), lower BMI (*p* = 0.023), and higher gravidity (*p* < 0.001), reflecting intersecting environmental, biological and socioeconomic vulnerabilities. Maternal Pb exposure in Kabwe remains alarmingly high, especially in mining-proximal, low-resource communities, underscoring a critical environmental injustice. Given potential for Pb to cross the placenta and documented links to negative birth outcomes, urgent interventions, including regular maternal screening and policy reform, are needed to potentially protect the health of the unborn baby and break cycles of intergenerational toxicity.

## Background

Lead (Pb) exposure remains one of the most widespread heavy metals in the environment, with adverse health effects and is particularly harmful to pregnant women and children. There is no safe level for Pb, as even low levels can affect the fetus and cause neurodevelopmental impairment (Naranjo et al. 2020). Lead has well-documented health effects in pregnancy, as evidenced in several studies. During pregnancy, lead crosses the placenta, entering fetal circulation and impairing neurodevelopment, growth, and pregnancy outcomes (RÍsovÁ 2019). Studies conducted previously have linked prenatal Pb exposure to low birth weight and preterm birth (Santana et al. 2023), as well as impaired cognitive functions in children (Zhang et al. 2015; Taylor et al. 2016; Merced-Nieves et al. 2022). Mobilization of Pb from the bone during periods of physiological stress, such as pregnancy, becomes an endogenous source of exposure that further compounds these risks (RÍsovÁ 2019). The authors indicate that lead crosses the placenta, entering fetal circulation and impairing neurodevelopment, growth, and pregnancy outcomes. Previous studies have linked prenatal Pb exposure to low birth weight and preterm birth (Santana et al. 2023) as well as impaired cognitive functions in children (Zhang et al. 2015; Taylor et al. 2016; Merced-Nieves et al. 2022). Mobilization of Pb from the bone during periods of physiological stress, such as pregnancy, becomes an endogenous source of exposure that further compounds these risks (RÍsovÁ 2019).

Emerging evidence from systematic reviews and meta-analyses highlights that maternal lead exposure is not only neurotoxic to the developing fetus but also linked with several adverse pregnancy outcomes beyond previously recognized endpoints including hypertensive disorders, preterm birth, and impaired fetal growth even at low blood lead levels (Habibian et al. 2022; Vigeh et al. 2023; Ou et al. 2025). A recent systematic review found consistent associations between elevated maternal BLLs and preeclampsia, gestational hypertension, and reduced anthropometric outcomes at birth, with toxic effects evident at concentrations below 5 µg/dL, emphasizing that no level of Pb is safe during pregnancy (Aliche et al. 2025). Complementing this, a large meta-analysis which focused on hypertensive disorders of pregnancy and reported that women with preeclampsia had more than twice the mean BLLs of women with normal blood pressure, thereby highlighting the role of lead in vascular pathology during gestation (Vigeh et al. 2025). Additional meta-analytic evidence links high maternal lead exposure with increased risks of preterm birth and small-for-gestational-age infants, reinforcing the relevance of Pb in adverse reproductive outcomes (Ou et al. 2025).

Crucially, socioeconomic and environmental determinants amplify maternal lead exposure and its effects. These characteristics of pregnant mothers provide essential context for understanding how lead exposure may influence maternal and fetal health (Montgomery and Mathee 2005; Perkins et al. 2014). The authors explain that in urban settings, lower socioeconomic status and older housing may be associated with higher Pb burdens in children and adults alike, reflecting structural inequities in exposure distribution. A study in the USA found that higher maternal BLLs were associated with lower household income and lower educational attainment, demonstrating that socioeconomic disadvantage corresponds with greater lead exposure even at low exposure levels (Ishitsuka et al. 2020). Although much of this research focused on childhood exposure, the same social determinants are relevant to pregnant women, as low-income communities often contend with multiple sources of contamination, limited access to prenatal care and screening, and higher nutritional vulnerabilities that can biologically increase Pb absorption.

More studies affirm that variations in sociodemographic factors such as education, occupation, location, and socioeconomic status highlight potential sources of vulnerability and differential exposure risk, which are critical for identifying at-risk populations (Taylor et al. 2016; Bose-O’Reilly et al. 2018; O’Connor et al. 2018; Yabe et al. 2018; Yamada et al. 2020). In another study, a systematic review of blood lead in women of childbearing age in sub-Saharan Africa highlighted higher BLLs in women from lower socioeconomic backgrounds, with residential proximity to contamination and limited resources being key risk factors in maternal exposure (Bede-Ojimadu et al. 2018).

Poor nutrition, which is more common in low-income settings, further multiplies susceptibility as women with lower socioeconomic status and poor micronutrient intake tend to have higher lead burdens, whereas diets richer in micronutrients such as calcium are associated with reduced lead (Cubello et al. 2023). Body mass index (BMI) is a key indicator of maternal nutritional status known to affect pregnancy outcomes (Maldonado-Cedillo et al. 2016). One of the vital nutrients is iron, which is prevalent in low-income settings and whose deficiency may adversely affect maternal health and exacerbate lead exposure. There is a bi-directional relationship between lead and anemia, where lead disrupts heme synthesis, while iron deficiency enhances Pb absorption, adversely affecting fetal growth and cognition (Bellinger 2005). Another factor that may influence lead absorption and exposure in pregnant women is gravidity. Studies show that maternal bone lead may account for a significant fraction of circulating lead during gestation and has been cited as an independent risk factor for fetal neurotoxicity (Gomaa et al. 2002). Although direct studies relating to gravidity and lead exposure are limited, evidence suggests that repeated pregnancies may influence maternal lead mobilization due to repeated bone resorption (Gulson et al. 1997, 2003). Together, these findings underscore the need to consider both biological and contextual factors when assessing the status and resulting impact of maternal lead exposure, ultimately informing strategies to protect fetal health in heavily contaminated settings like Kabwe, Zambia.

In Zambia, Pb contamination has been reported in Kabwe, near the former lead-zinc mine and is currently recognized globally as one of the world’s most polluted sites (Baieta et al. 2021). Previous studies have shown extremely high blood lead levels (BLLs) in children from Kabwe townships, which were attributed to contaminated soils (Yabe et al. 2015, 2020). The relationship between BLLs and soil Pb concentrations highlights the strong contribution of environmental contamination to maternal exposure. This highlights the risk posed by living in areas with historically contaminated soil, where lead can be ingested or inhaled through dust, particularly in residential zones near mining sites. Nevertheless, there is inadequate information on the status and resulting impact of high Pb exposure on pregnant women, despite being a highly vulnerable group.

The present study, therefore, investigated BLLs among pregnant women in selected townships of Kabwe District with varying risks of lead exposure. This study advances prior research on lead exposure by shifting the focus from the extensively documented children's lead burden in Kabwe to the comparatively under examined risks faced by pregnant women and their unborn babies. By specifically assessing maternal blood lead levels in relation to residential proximity to legacy mining contamination, the study provides direct evidence of in utero exposure pathways in a highly contaminated setting. In addition, by integrating socioeconomic indicators, it moves beyond environmental measurement alone to demonstrate how structural inequities shape exposure risk. This environmental justice perspective highlights that lead exposure is not only a toxicological issue but also a function of poverty, land use patterns, and historical mining practices. This study integrates spatial and biological exposure data and situates the findings within an environmental justice framework in a low-resource mining context, thereby linking maternal health, fetal vulnerability, and social disadvantage in a single analytical framework.

### Hypotheses 

This study therefore examines the hypothesis that environmental and socioeconomic factors are associated with maternal BLLs in a legacy-mining context.

The H0_1_ is that there is no association between residential proximity to legacy mining contamination and maternal BLLs, while the alternative hypothesis H1_1_ is that maternal BLLs are inversely associated with distance from contamination sources.

H0_2_ is that socioeconomic status (SES) is not associated with maternal BLLs and H1_2_ is that lower SES is associated with higher maternal BLLs independent of spatial proximity.

H0_3_ is that SES does not modify the relationship between proximity and maternal BLLs and H1_3_ is that the association between proximity and maternal BLLs is stronger among women of lower SES.

H0_4_ is that maternal biological characteristics, i.e., gravidity, anemia status, and BMI, are not associated with maternal BLLs, and H1_4_ is that maternal biological characteristics are associated with maternal BLLs. These hypotheses are grounded in environmental exposure theory, lead toxicokinetics, and environmental justice frameworks, and in the study, they are tested using spatial and multivariable regression analyses to clarify the interrelationships among environmental contamination, structural inequities, and fetal health risk in Kabwe, Zambia.

## Methods

### Study design

This was a quantitative comparative cross-sectional study, where two groups of pregnant women attending antenatal services in designated health centers in the vicinity of and away from the Pb mine were enrolled to compare varying degrees of lead exposure through measurement of BLLs. The study design was selected to assess and compare maternal BLLs between two groups at a single point in time. This measured the varying degrees of exposure between the two populations and allows for direct comparison between pregnant women residing near the legacy Pb mine and those living farther away. By enrolling participants from antenatal clinics in both high-exposure and lower-exposure areas, the study efficiently captured spatial differences in environmental exposure to Pb.

### Study area and sampling location

The study was undertaken in Kabwe District of Zambia. Kabwe is the provincial capital of Zambia’s Central Province, located at approximately 28°26′E and 14°27S. It is the fourth-largest town in Zambia, with a population of about 230,000 inhabitants and covering an area of 1,547 km. In Kabwe, where formal employment opportunities are limited, many women rely on informal work such as street vending, artisanal mining, stone crushing, or trading in local markets. Health Facilities from the selected/designated study townships in Kabwe District were purposively selected to align with the research objectives. The key selection criteria for the health facilities included the availability of maternity services and the distance of each health center from the mine. This approach facilitated a comparative analysis between health centers located near the mine and those farther away. Specifically, Kasanda (1.5 km) and Makululu (2.5 km) are located close to the mine, in the windward direction, while Katondo (4.5 km) and Mahatma Gandhi (4.5 km) were located farther away, towards the northeastern part of the mining area. This distribution of health centers provided a balanced representation of populations potentially exposed to varying levels of lead contamination.

### Sample size

**Fig. 1:**
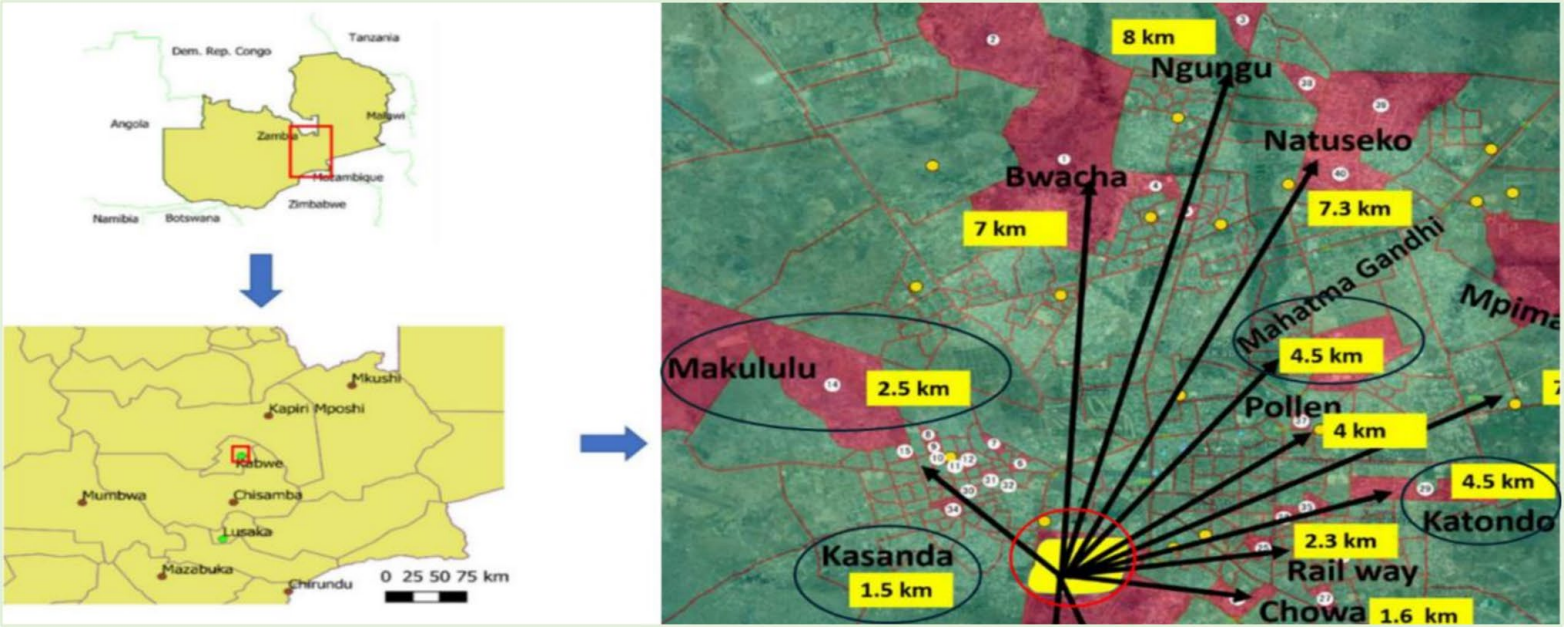
The study site in Kabwe, Zambia, and the townships where pregnant mothers reside. The top left panel shows the map of Zambia and the map of Central Province (insert). The right panel illustrates the location of the study area in Kabwe, Zambia (source: (Yabe et al. 2020). The map also indicates the health centers and their respective distances from the Pb-Zn mine: Kasanda (1.5 km), Katondo (4.5 km), Makululu (2.5 km), and Mahatma Gandhi (4.5 km). Health centers are marked with black circles, while the Pb-Zn mine is represented by a red circle

The sample size calculation was based on the quantitative comparative cross-sectional study design to determine birth outcomes (average birthweight and gestational age) among pregnant women with high blood lead exposure and those with low blood lead exposure. The statistical parameters considered were 80% statistical power, 95% confidence level, a minimum difference of 160 g in birthweight, standard deviation of 520 g (Perkins et al. 2014), design effect of 1.5, and a 5% significance level. This translated to a minimum sample of 498 women. In the study, 510 women were enrolled, from whom blood samples were collected and analyzed. This resulted in 223 with high lead exposure and 287 with low lead exposure. The final sample of mothers according to townships is in the Appendix (Fig. 1).

### Sampling technique

The study involved a two-stage sampling where four Health Facilities were purposively sampled and the pregnant women were sampled using Simple Random Sampling from the Health facilities’ antenatal attendance register.

#### Sampling of health facilities

Health centers were purposively sampled to facilitate a comparative analysis between pregnant women from Health facilities located near the Lead –Zinc mine and those situated further away. Other criteria were the provision of maternity services in these Health Facilities. This provided a balanced representation of populations potentially exposed to varying levels of lead contamination.

### Sampling of pregnant mothers

The study population comprised 510 pregnant women who were recruited from Kasanda, Makululu, Katondo, and Mahatma Gandhi. Following Simple Random Sampling of pregnant women from a sampling list based on the Antenatal Register, they were enrolled in the study after obtaining informed consent, in accordance with the protocol approved by ERES Converge (Ref. No. 2022-Sep-029).

### Data collection tools

Data collection tools included a structured questionnaire for sociodemographic information and a laboratory blood sample collection form.

### Data collection assistants

Trained midwives working in the Maternal and Child Health Clinics in the study sites collected sociodemographic information and blood samples. The midwives were oriented in the administration of the questionnaire and in how much of the blood sample should be collected.

### Collection of sociodemographic and clinical information

Social and demographic information was collected from pregnant women using a questionnaire administered by qualified midwives working at the designated study sites. Information collected included maternal age, height, weight, education (categorized as primary, secondary, and tertiary), and occupation.

Other information collected was BMI, hemoglobin levels (Hb), self-reported maternal smoking during pregnancy, self-reported maternal alcohol consumption during pregnancy and any medical history that may affect pregnancy outcome, number of pregnancies and their outcomes and history of hypertension, and years of stay in the study area.

### Blood sample collection

Blood samples were collected in accordance with the Centres for Disease Control and Prevention guidelines for handling blood samples, with minor modifications to suit local conditions.

Trained midwives collected approximately 5 mL of venous blood from the pregnant women. The samples were placed in sterile BD Vacutainer® Lithium Heparin (95 USP Units, NJ 07417, USA) tubes and securely sealed in Ziploc plastic bags to prevent contamination. All collected samples were temporarily stored at −20°C in a freezer at Kasanda Clinic. For subsequent analysis, the samples were transported in cooler boxes packed with ice to the Toxicology Laboratory, University of Zambia (UNZA) in Lusaka, Zambia.

### Sample preparation and digestion

All laboratory materials and instruments used for metal extraction were pre-cleaned with 2% nitric acid (HNO_3_) (HiMedia Laboratories Pvt. Ltd, India), thoroughly rinsed with double-deionized water (DDW) from a Milli-Q Element System (18 MΩ·cm, Millipore®, Milford, MA, USA), and oven-dried at 50 °C to eliminate any potential contaminants.

Lead concentrations in the samples were determined following the method described by Tembo et al. (2006) with minor modifications. Blood samples were homogenized using a vortex mixer. Approximately 300 µL of each blood sample was transferred into pre-cleaned Berghof digestion vessels (DAP-60K, Eningen, Germany). To each sample, 5mL of 30% nitric acid (diluted from 69% HNO_3_) was added. Metal extraction was carried out using a closed, optimized microwave digestion system (Berghof SpeedWave® ENTRY, Eningen, Germany) under automated temperature-controlled and pressure-controlled conditions for 31 min.

The digested samples were then cooled for 20 min, transferred into 15-mL Falcon tubes (Fisher Scientific, Waltham, MA, USA), and diluted to a final volume of 10 mL with DDW. To ensure complete homogenization, the tubes were inverted 10 times. Blank samples were prepared in parallel with the blood samples to maintain consistency in digestion conditions.

To prevent contamination, digestion vessels were thoroughly cleaned after each cycle using 60% nitric acid before reuse.

### Metal analysis and sample quality control

Lead (Pb) concentrations were quantified using Polarised Zeeman Graphite Furnace Atomic Absorption Spectrophotometry (GFAAS) employing a Hitachi ZA-2010 model (High Technologies Corporation, Tokyo, Japan). The ZA-2010 series represents an advanced generation of atomic absorption spectrometers designed to deliver exceptional accuracy and sensitivity through the application of polarized Zeeman background correction technology. This system enhances analytical reliability and precision, offering superior detection capabilities compared to conventional atomic absorption instruments.

Instrumental conditions and analytical parameters were optimized for lead determination and are summarized in Table 4 (Appendix).

For quality control, all the chemicals, reagents, and standard solutions used were of analytical grade, and DDW was used throughout the sample processing and analysis. The BLLs were measured using AAS. The detailed operating set conditions are outlined in Table 1. Analytical quality control was performed using the DOLT-5 (fish liver, National Research Council Canada, Ottawa, Canada) certified reference material. Replicated analysis of the reference materials showed a good recovery rate, ranging from 92.3 to 95.1% (Table 5 (Appendix)).

**Table 1:** Comparison of sociodemographic characteristics by level of blood lead exposure

Sociodemographics	Overall ***N*** = 510 *n* (%)	Level of lead exposure		
		High ***N*** = 223 *n* (%)	Low *N* = 287 *n* (%)	*p*-value
Maternal age in years, median (IQR)	25 (20, 30)	25 (20, 31)	25 (20, 30)	0.868
Years stayed in the area, median (IQR)	9 (4, 18)	10 (4, 10)	8 (4, 8)	**0.007**
Level of education * Primary* * Secondary* * Tertiary*				0.091
	122 (34.0)	69 (35.9)	53 (31.7)	
	222 (61.8)	119 (62.0)	103 (61.7)	
	15 (4.2)	4 (2.1)	11 (6.6)	
Employment status				** < 0.001**
* Formal*	23 (4.6)	0 (0.0)	23 (8.2)	
* Informal*	94 (18.8)	41 (18.7)	53 (18.8)	
* Unemployed*	384 (76.6)	178 (81.3)	206 (73.0)	
Current alcohol consumption				**0.011**
* No*	470 (93.1)	211 (96.3)	259 (90.6)	
* Yes*	35 (6.9)	8 (3.7)	27 (9.4)	
Current smoking status				**0.021**
* No*	459 (90.7)	193 (87.3)	266 (93.3)	
* Yes*	47 (9.3)	28 (12.7)	19 (6.7)	

The method detection limit (MDL) for Pb was 0.012 μg/g, and the method quantification limit (MQL) was 0.045 μg/g. The Pb concentrations were expressed as μg/dL. 

To ensure data integrity and prevent cross-contamination in subsequent measurements, a 2% HNO_3_ solution was used after every five samples to wash the sample collection probe.

Additionally, a blank sample was analyzed after every nine samples to monitor and control any potential contamination during the analytical process. The analysis was strategically conducted, beginning with samples from Health Facilities located further from the mine, presumed to have lower Pb concentrations and concluding with Makululu, expected to exhibit higher Pb levels due to its proximity to the mining area.

### Data management

After collection, the data was entered into the Excel spreadsheet and kept in a secure manner on a separate external hard drive. During data collection and laboratory analysis, the names of mothers in all study areas were de-identified using codes.

### Statistical analysis

Data were cleaned, coded, and analyzed using R software version 4.5.1. Categorical variables were summarized using frequencies and percentages, whereas numeric variables were summarized as medians and interquartile ranges due to skewness. The distribution of numeric variables was assessed statistically and graphically, using box-plots, histograms, and Q-Q plots. Blood lead levels (BLLs) were categorized as high (> 3.5 μg/dL) or low (≤ 3.5 μg/dL) based on the CDC reference value (Ruckart 2021). Although BLL is inherently continuous, dichotomization was performed to align with established public health thresholds and to enhance interpretability in terms of risk classification and policy relevance. Additionally, sensitivity analyses were performed, in which we examined BLL as a continuous variable, and the direction of associations remained consistent for all variables (results not presented). Comparisons of levels of lead exposure across independent variables (demographic and clinical factors) were conducted using the chi-square test, or Fisher’s exact test for categorical variables, and the Wilcoxon rank sum test, or the Kruskal–Wallis test for numerical variables. To determine maternal BLLs, overall proportions and median summaries were presented, and comparisons across location and maternal lead exposure were performed using non-parametric statistics.

## Results

### Sociodemographic characteristics by level of blood lead exposure

Results in Table 1 show that women in the study had a median age of 25 years (IQR: 20–30 years) and had stayed in the current area of residence for 9 years (IQR: 4–18 years). Most women (61.8%, 222) attained secondary-level education, and the majority (76.6%, 384) were not in formal employment. Current alcohol intake (6.9%, 35) and cigarette smoking (9.3%, 47) were less prevalent among study participants.

Participants with high Pb exposure were found to have stayed in their current area of residence longer (10 years, IQR: 4–10) with a statistical significance of p = 0.007 than those with low Pb exposure (8 years, IQR: 4–8). Similarly, the proportions of Pb exposure showed significant variations across levels of employment (*p* < 0.001), alcohol consumption (*p* = 0.011), and smoking (0.021), with higher levels of Pb exposure more prevalent among the unemployed (81.3%, 178), and among current smokers (12.7%, 28) compared to their counterparts.

### Maternal clinical characteristics by level of blood lead exposure

Table 2 shows that study participants had a median BMI of 25 kg/m^2^ (IQR: 22, 27), gravidity of 2 (IQR: 0, 3), and Hb level of 12 g/dL (IQR: 11, 13). The majority of women reported no history of hypertension (97%, 480) or other chronic conditions (97.8%, 490). Significant differences were observed in BMI (*p* = 0.023) and gravidity (*p* < 0.001) across levels of lead exposure, with high lead-exposed women showing a lower median BMI (24 kg/m^2^) compared to those with low exposure (25 kg/m^2^). Gravidity appeared higher among women with high lead exposure (2 pregnancies) than those with low exposure (1 pregnancy).

**Table 2:** Comparison of maternal clinical characteristics by level of blood lead exposure

Clinical characteristics		Level of lead exposure		*p*-value
	Overall ***N*** = 510 *n* (%)	High *N* = 223 *n* (%)	Low *N* = 287 *n* (%)	
Body mass index, *median (IQR)*	25 (22.0, 27.0)	24 (21.9, 26.8)	25 (22.0, 28.0)	0.023
Gravid, *median (IQR)*	2 (0.0, 3.0)	2 (0.0, 3.0)	1 (0.0, 2.0)	** < 0.001**
Hemoglobin level, *median (IQR)*	12 (11.0, 13.0)	12 (10.9, 12.5)	12 (11.1, 13.0)	0.079
History of hypertension				0.442
* No*	480 (97.0)	208 (96.3)	272 (97.5)	
* Yes*	15 (3.0)	8 (3.7)	7 (2.5)	
History of other chronic conditions				0.763
* No*	490 (97.8)	215 (98.2)	275 (97.5)	
* Yes*	11 (2.2)	4 (1.8)	7 (2.5)	

### Levels of lead among pregnant women living in selected townships of Kabwe District

As shown in Table 3, pregnant women in the study had a median blood lead concentration of 3.2 µg/dL (IQR: 1.7, 6.0). Overall, 43.7% (223) of participants had high BLLs, with the highest BLLs of 133 µg/dL. Significant differences in both medians and proportions were observed across the four townships (*p* < 0.001), with pregnant women from Makululu having the highest median BLL (6.5 µg/dL, IQR: 4.6, 8.4), followed by those from Kasanda (3.4 µg/dL, IQR: 2.4, 5.6). Lower concentrations were observed in Katondo (1.8 µg/dL, IQR: 1.2, 2.9) and Mahatma Gandhi (1.6 µg/dL, IQR: 1.1, 2.5). Similarly, the proportion of pregnant women with high BLL was highest in Makululu (84.4%, 151) and Kasanda (48.1%, 51), compared to Katondo (10.0%, 10) and Mahatma Gandhi (8.8%, 11).

**Table 3:** Levels of lead among pregnant women living in selected townships of Kabwe District

Characteristics	Overall *N* = 510 *n* (%)	Township of residence				*p*-value
		Makululu *N* = 179 *n* (%)	Kasanda *N* = 106 *n* (%)	Katondo *N* = 100 *n* (%)	Mahatma Gandhi *N* = 125 *n* (%)	
Blood lead concentration, *median (IQR)*	3.2 (1.7, 6.0)	6.5 (4.6, 8.4)	3.4 (2.4, 5.6)	1.8 (1.2, 2.9)	1.6 (1.1, 2.5)	** < 0.001**
Blood lead levels						** < 0.001**
* High (***> ***3.5 µg/dL)*	223 (43.7)	151 (84.4)	51 (48.1)	10 (10.0)	11 (8.8)	
* Low (***< ***3.5)*	287 (56.3)	28 (15.6)	55 (51.9)	90 (90.0)	114 (91.2)	

**Fig. 2:**
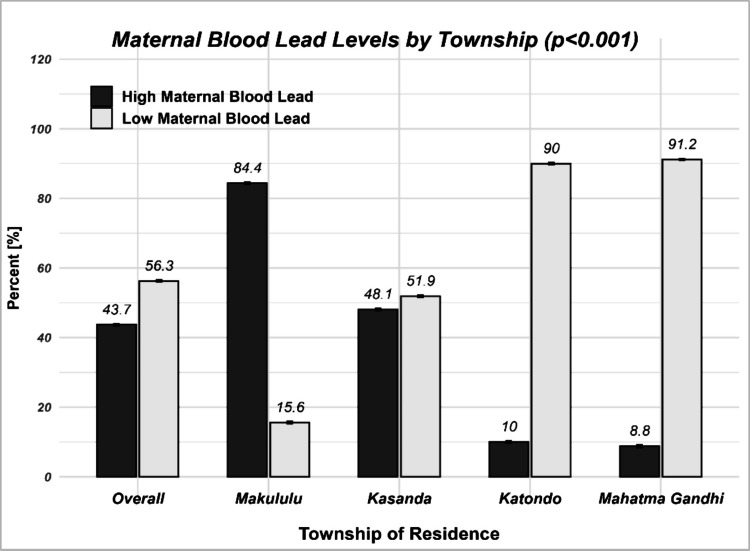
The overall proportion of BLLs, according to the township

As shown in Fig. 2, participants from the four townships had varying levels of BLLs, with Makululu and Kasanda recording the highest proportion of elevated levels, compared to Katondo and Mahatma Gandhi townships.

## Discussion

### Blood lead levels among pregnant women

The study revealed substantial spatial variation in BLLs across Kabwe District, with an overall median of 3.2 µg/dL (IQR: 1.7–6.0) and 43.7% of pregnant women exceeding exposure thresholds, confirming persistent contamination from legacy mining and smelting activities (Tembo et al. 2006; Bose-O’Reilly et al. 2018; Yabe et al. 2020). Makululu showed the highest BLLs (6.5 µg/dL; 84.4%), followed by Kasanda (3.4 µg/dL; 48.1%), both are low-income areas near the former mine with poor housing and inadequate access to Pb remedial measures, and limited remediation access. In contrast, Katondo (1.8 µg/dL) and Mahatma Gandhi (1.6 µg/dL) recorded lower exposures, consistent with their distance from the contamination epicenter and better housing conditions (Tembo et al. 2006; Yabe et al. 2013; Bose-O’Reilly et al. 2018). These differences (*p* < 0.001) highlight the spatial heterogeneity of Pb exposure and its socioeconomic dimensions. Despite lower median BLLs than earlier child-focused studies (Yabe et al. 2020; Bose-O’Reilly et al. 2018), nearly half of the study participants remain at risk, underscoring ongoing maternal-fetal health concerns (Hertz-Picciotto et al. 2000; Gomaa et al. 2002). These findings call for strengthened remediation, biomonitoring, and community interventions to reduce intergenerational lead toxicity in Kabwe.

The present study findings mirror global concerns of Pb exposure, where Pb poisoning remains a major economic and public health burden, costing over US$6 trillion, equivalent to about 6.9% of the global gross domestic product (Larsen and Sánchez-Triana 2023). For Kabwe, just like other contaminated sites, Pb stored in maternal bones is remobilized during pregnancy, directly exposing the fetus (RÍsovÁ 2019) and contributing to adverse outcomes such as low birth weight (Aliche et al. 2025; Wessel 2025). Makululu’s median BLLs nearly double the CDC reference value of 3.5 µg/dL (Ruckart 2021), posing urgent fetal health risks. These exposures reflect structural inequities, where women are affected not by choice but by environmental and socioeconomic disadvantage.

### Maternal sociodemographic information

The median age of mothers was 25 years, showing no significant link between age and BLLs, consistent with studies in Mexico and South Africa, where age had little effect once housing and occupation were taken into account. Women from Makululu, near the former Pb mine, had the highest BLLs, aligning with previous findings that attribute higher exposure in Makululu and Kasanda to proximity to the mine. Education levels showed no significant difference among the exposed women, although tertiary education was more common among those with lower BLLs, echoing global evidence that links education to better living conditions and less exposure. However, in highly contaminated areas like Makululu, the protective benefit of education seemed reduced, indicating that environmental contamination can diminish socioeducational advantages.

Most women in the study were unemployed, and higher BLLs were observed among them compared to those in formal employment. This aligns with global evidence showing that lower socioeconomic status is linked to higher exposure to environmental toxicants, including lead, due to poor housing, limited mobility, and dependence on contaminated local resources (Hiwatari et al. 2024). Income and residence in contaminated areas strongly influence Pb exposure and adverse birth outcomes (Bellinger 2005; O’Connor et al. 2018), as poor families often inhabit polluted environments, facing greater health risks, higher healthcare costs, and limited access to quality education (Yoshii et al. 2020). The study found the strongest association between BLLs and place of residence, with 67.7% of women from Makululu, an area characterized by poor housing, unpaved yards, and proximity to mine waste, showing high exposure compared to women from Katondo and Mahatma Gandhi. These findings underscore how socioeconomic disadvantage amplifies vulnerability to environmental contamination, reinforcing the intersection between poverty and public health inequality.

The findings in the present study also align with previous reports in Kabwe (Bose-O’Reilly et al. 2018; Yamada et al. 2020), where communities near the mine with inadequate infrastructure face the highest lead risks and this has also been reported in other studies (de Barberin-Barberini et al. 2025).

The finding that participants with high Pb exposure had resided significantly longer in their current area with a median of 10 years (IQR: 4–10 years) compared to those with lower exposure with a median of 8 years (IQR: 4–8 years) (*p* = 0.007) suggests a cumulative exposure effect consistent with the environmental context of Kabwe. In communities situated near legacy mining contamination, prolonged residence likely increases the duration and intensity of contact with contaminated soil, household dust, and locally grown food products. Given that lead persists in the environment and does not degrade over time, extended habitation in contaminated areas may contribute to sustained body burden through chronic low-level exposure. This association underscores the importance of residential stability as a determinant of maternal BLLs. Women who have lived longer in contaminated neighborhoods may have experienced repeated exposure over several years, including prior to pregnancy. Importantly, Pb stored in maternal bone from past exposure can be mobilized during pregnancy due to increased calcium demands, thereby elevating circulating BLLs and increasing the risk of transplacental transfer to the fetus. This mechanism may partially explain the higher exposure observed among long-term residents. The findings also reflect underlying socioeconomic disparities. Households with limited financial resources may have reduced mobility and fewer opportunities to relocate away from contaminated environments, perpetuating intergenerational exposure risks. In the case of Kabwe, where historical mining activities have left widespread environmental contamination, prolonged residence in high-risk zones may represent both environmental and social vulnerability. Combined, the findings suggest that maternal BLLs in Kabwe are shaped by a combination of geographic proximity to legacy mining contamination, cumulative duration of environmental exposure, and socioeconomic immobility. This highlights the need for geographically targeted interventions including environmental remediation, routine antenatal lead screening in high-risk townships, and social support mechanisms to break the cycle of exposure in communities closest to the former mine.

### Clinical characteristics of pregnant women

The study found that women with lower BMI had higher BLLs compared to those with higher BMI, suggesting that undernutrition increases vulnerability to lead absorption. WHO classifies undernutrition as a BMI below 18.5 and obesity as above 40 (Zaballa et al. 2012), making BMI a useful indicator of nutritional status in pregnancy. Poor nutrition can increase lead absorption by increasing gastrointestinal uptake and reducing competition with essential minerals such as calcium and iron (Ettinger et al. 2009). This creates a dual risk, where undernutrition not only impairs fetal growth but also enhances maternal and fetal Pb exposure. Experimental studies have confirmed that maternal undernutrition and iron deficiency elevate fetal lead uptake and compromise development (Maldonado-Cedillo et al. 2016). Overall, these findings emphasize that improving maternal nutrition is essential to reducing lead toxicity risks during pregnancy.

In the present study, gravidity was one of the maternal characteristics assessed in relation to BLLs. Women with two or more previous pregnancies had higher BLLs than those with one or no prior pregnancies. This likely reflects increased bone Pb reserves due to chronic exposure to lead, as well as increased bone resorption during pregnancy. This was in contrast to other studies, where lower BLLs were reported in women who had more pregnancies than those with one or no history of pregnancy, implying that the greatest risk of lead toxicity lies with the first pregnancy (Vigeh et al. 2011). Maternal bone acts as a major reservoir for stored lead, which can be released into the bloodstream during pregnancy due to increased calcium demand. This process results in endogenous lead exposure, and with each successive pregnancy, more lead is mobilized from bone stores into the maternal circulation, where it can cross the placenta and expose the fetus. Consequently, women with a higher number of previous pregnancies (high-gravidity women) may experience greater cumulative lead release over time, increasing both their own BLLs and the risk of fetal exposure and adverse implications for the fetus (Gulson et al. 1997, 2003). As such, with chronic exposure in contaminated areas like Kabwe, the risk of fetal lead exposure in women who have had more pregnancies may be higher (Jelliffe-Pawlowski et al. 2006).

Given that the study also assessed hemoglobin levels to evaluate anemia among pregnant women, the results obtained revealed that pregnant women with high BLLs had hemoglobin levels of IQR of 10.9 (12g/dL). The WHO (2024) defines anemia in pregnancy as being less than 11.0g/dL and not less than 12.0 dL in non-pregnant women. Although no statistically significant difference was found between high and low lead groups, the lower Hb values among women with higher BLLs remain a concern, as even mild anemia can worsen hypoxia and nutritional stress, contributing to poor birth outcomes (Yadav et al. 2020).

The present study found no significant difference in hypertension between women with high and low BLLs, likely due to the young median age of 25 years, as hypertension typically develops later in life; however, long-term exposure to lead has been associated with elevated blood pressure. (Navas-Acien et al. 2007). Nearly half of the pregnant women tested had elevated BLLs, with a median of 3.2 µg/dL, an alarming finding given that no safe lead level exists.

Addressing Kabwe’s lead crisis requires an integrated approach that combines environmental clean-up with social and behavioral interventions targeting poverty, education, and risky practices to reduce maternal and child exposure. Immediate priorities include maternal biomonitoring, community education, and long-term environmental remediation through stricter mining regulation, improved housing, and infrastructure investment. Ultimately, protecting maternal and child health in Kabwe demands sustained, multisectoral action to mitigate the generational lead exposure.

### Limitations

The main limitation in this study relates to exposure measurement time. Blood lead levels were assessed at a single time point of pregnancy during enrollment using whole blood samples. Given the complex kinetics of Pb, including long-term storage in the bones and differential distribution between plasma and whole blood, a single measurement may not fully have captured cumulative exposure or exposure during critical gestational windows. Furthermore, the absence of bone Pb measurement or repeated measurements may not have accurately quantified the BLLs in the women in relation to specific exposure windows during pregnancy. Another limitation was the structured questionnaire, which was not pre-tested and validated in the target population thereby limiting the accuracy and appropriateness of the collected data.

## Conclusion

This study reveals that a significant proportion of pregnant women in selected townships of Kabwe have elevated BLLs, with higher exposures observed in communities located closer to the former mining site. These findings highlight the persistent impact of legacy mining contamination on maternal exposure and stress the spatial dimension of environmental risk. Given the well-known toxicity of lead during pregnancy and its potential adverse developmental outcomes, strengthened environmental remediation, exposure monitoring, and integration of maternal screening into public health strategies are urgently required. Addressing ongoing contamination in Kabwe remains critical to reducing exposure and mitigating long-term environmental and intergenerational health consequences.

## Data Availability

All the data included in the current study are available on request.

## Appendix

Table 4 and 5.

**Table 4:** Sample distribution by distance from the mine

No.	Name of health facility	Proximity to the mines	Sample required
1	Kasanda Urban Health Centre	1.5 km	106
2	Katondo Urban Health Centre	4.5 km	100
3	Makululu Urban Health Centre	2.5 km	179
4	Mahatma Ghandi Memorial Urban Health Centre	4.5 km	125
	**Total**		**510**

**Table 5:** Z-2000 series—GFAAS method parameters

Instrument parameter	Settings
Unit	µg/L
STD replicate	2
Decimal place	2
Sample replicate	2
Reslope interval	10
Injection volume (µL)	20
Auto-sampler	On
Conc. times	1
Rinse replicate	3
Injection speed	4
Cleaning times (s)	5
RSD limit (%)	10
Curve order	Linear
Background correction	Auto
Argon pure (%)	99.9

## Declarations

### Clinical trial number

Clinical trial number is not applicable since the study was not a clinical trial.

## References

[CR1] Aliche KA, Ikewuchi C, Diorgu FC (2025) The impact of maternal lead exposure on pregnancy outcomes: a systematic review. IPS J Public Health 5:226–236 10.1177/11786302251327535 PMC12033612 40290266

[CR2] Baieta R, Mihaljevič M, Ettler V, Vaněk A, Penížek V, Trubač J, Kříbek B, Ježek J, Svoboda M, Sracek O (2021) Depicting the historical pollution in a Pb–Zn mining/smelting site in Kabwe (Zambia) using tree rings. J Afr Earth Sci 181:104246. 10.1016/j.jafrearsci.2021.104246

[CR3] Bede-Ojimadu O, Amadi CN, Orisakwe OE (2018) Blood lead levels in women of child-bearing age in Sub-Saharan Africa: a systematic review. Front Public Health. 10.3389/fpubh.2018.00367 10.3389/fpubh.2018.00367 PMC6305709 30619808

[CR4] Bellinger DC (2005) Teratogen update: lead and pregnancy. Birth Defects Res A Clin Mol Teratol 73:409–420. 10.1002/bdra.20127 15880700 10.1002/bdra.20127

[CR5] Bose-O’Reilly S, Yabe J, Makumba J, Schutzmeier P, Ericson B, Caravanos J (2018) Lead intoxicated children in Kabwe, Zambia. Environ Res 165:420–424. 10.1016/j.envres.2017.10.024 29089102 10.1016/j.envres.2017.10.024

[CR6] Cubello J, Peterson DR, Wang L, Mayer-Proschel M (2023) Maternal iron deficiency and environmental lead (Pb) exposure alter the predictive value of blood Pb levels on brain Pb burden in the offspring in a dietary mouse model: an important consideration for cumulative risk in development. Nutrients 15:4101 37836385 10.3390/nu15194101 PMC10574741

[CR7] de Barberin-Barberini H, Jouve E, Dubus J-C, Hadji K, Laporte R (2025) Fighting lead poisoning: effective conditions for home-based education, housing remediation, and relocation. Toxics 13:552. 10.3390/toxics13070552 40710997 10.3390/toxics13070552 PMC12298219

[CR8] Ettinger AS, Lamadrid-Figueroa H, Téllez-Rojo MM, Mercado-García A, Peterson KE, Schwartz J, Hu H, Hernández-Avila M (2009) Effect of calcium supplementation on blood lead levels in pregnancy: a randomized placebo-controlled trial. Environ Health Perspect 117:26–31. 10.1289/ehp.11868 19165383 10.1289/ehp.11868 PMC2627861

[CR9] Gomaa A, Hu H, Bellinger D, Schwartz J, Tsaih S-W, Gonzalez-Cossio T, Schnaas L, Peterson K, Aro A, Hernandez-Avila M (2002) Maternal bone lead as an independent risk factor for fetal neurotoxicity: a prospective study. Pediatrics 110:110–118. 10.1542/peds.110.1.110 12093955 10.1542/peds.110.1.110

[CR10] Gulson BL, Jameson C, Mahaffey K, Mizon K, Korsch M, Vimpani G (1997) Pregnancy increases mobilization of lead from maternal skeleton. J Lab Clin Med 130:51–62. 10.1016/S0022-2143(97)90058-5 9242366 10.1016/s0022-2143(97)90058-5

[CR11] Gulson BL, Mizon KJ, Korsch MJ, Palmer JM, Donnelly JB (2003) Mobilization of lead from human bone tissue during pregnancy and lactation—a summary of long-term research. Sci Total Environ 303:79–104. 10.1016/S0048-9697(02)00355-8 12568766 10.1016/s0048-9697(02)00355-8

[CR12] Habibian A, Abyadeh M, Abyareh M, Rahimi Kakavandi N, Habibian A, Khakpash M, Ghazi-Khansari M (2022) Association of maternal lead exposure with the risk of preterm: a meta-analysis. J Matern Fetal Neonatal Med 35:7222–7230. 10.1080/14767058.2021.1946780 34210236 10.1080/14767058.2021.1946780

[CR13] Hertz-Picciotto I, Schramm M, Watt-Morse M, Chantala K, Anderson J, Osterloh J (2000) Patterns and determinants of blood lead during pregnancy. Am J Epidemiol 152:829–837. 10.1093/aje/152.9.829 11085394 10.1093/aje/152.9.829

[CR14] Hiwatari M, Yamada D, Narita D, Hangoma P, Chitah B (2024) Toxic pollution and poverty: economic impacts of lead (Pb) exposure on household welfare in Zambia. Ecol Econ 221:108209. 10.1016/j.ecolecon.2024.108209

[CR15] Ishitsuka K, Yamamoto-Hanada K, Yang L, Mezawa H, Konishi M, Saito-Abe M, Sasaki H, Nishizato M, Sato M, Koeda T, Ohya Y (2020) Association between blood lead exposure and mental health in pregnant women: results from the Japan environment and children’s study. Neurotoxicology 79:191–199. 10.1016/j.neuro.2020.06.003 32526257 10.1016/j.neuro.2020.06.003

[CR16] Jelliffe-Pawlowski L, Miles S, Courtney J, Materna B, Charlton V (2006) Effect of magnitude and timing of maternal pregnancy blood lead (Pb) levels on birth outcomes. J Perinatol 26:154–162. 10.1038/sj.jp.7211453 16453008 10.1038/sj.jp.7211453

[CR17] Larsen B, Sánchez-Triana E (2023) Global health burden and cost of lead exposure in children and adults: a health impact and economic modelling analysis. Lancet Planet Health 7:e831–e840. 10.1016/S2542-5196(23)00166-3 37714172 10.1016/S2542-5196(23)00166-3

[CR18] Maldonado-Cedillo BG, Díaz-Ruiz A, Montes S, Galván-Arzate S, Ríos C, Beltrán-Campos V, Alcaraz-Zubeldia M, Díaz-Cintra S (2016) Prenatal malnutrition and lead intake produce increased brain lipid peroxidation levels in newborn rats. Nutr Neurosci 19:301–309. 10.1179/1476830515Y.0000000003 25650657 10.1179/1476830515Y.0000000003

[CR19] Merced-Nieves FM, Chelonis J, Pantic I, Schnass L, Téllez-Rojo MM, Braun JM, Paule MG, Wright RJ, Wright RO, Curtin P (2022) Sexually dimorphic associations between prenatal blood lead exposure and performance on a behavioral testing battery in children. Neurotoxicol Teratol 90:107075. 10.1016/j.ntt.2022.107075 35108597 10.1016/j.ntt.2022.107075 PMC8957713

[CR20] Montgomery M, Mathee A (2005) A preliminary study of residential paint lead concentrations in Johannesburg. Environ Res 98:279–283. 10.1016/j.envres.2004.10.006 15910783 10.1016/j.envres.2004.10.006

[CR21] Naranjo VI, Hendricks M, Jones KS (2020) Lead toxicity in children: an unremitting public health problem. Pediatr Neurol 113:51–55. 10.1016/j.pediatrneurol.2020.08.005 33011642 10.1016/j.pediatrneurol.2020.08.005

[CR22] Navas-Acien A, Guallar E, Silbergeld EK, Rothenberg SJ (2007) Lead exposure and cardiovascular disease—a systematic review. Environ Health Perspect 115:472–482. 10.1289/ehp.9785 17431501 10.1289/ehp.9785 PMC1849948

[CR23] O’Connor D, Hou D, Ye J, Zhang Y, Ok YS, Song Y, Coulon F, Peng T, Tian L (2018) Lead-based paint remains a major public health concern: a critical review of global production, trade, use, exposure, health risk, and implications. Environ Int 121:85–101. 10.1016/j.envint.2018.08.052 30179767 10.1016/j.envint.2018.08.052

[CR24] Ou Y, Han J, Wei Y, Gao X (2025) Meta-analysis of maternal exposure to heavy metals (lead, cadmium, mercury, chromium) and adverse pregnancy outcomes. BMC Pregnancy Childbirth 26:7. 10.1186/s12884-025-08507-x 41316017 10.1186/s12884-025-08507-x PMC12763927

[CR25] Perkins M, Wright RO, Amarasiriwardena CJ, Jayawardene I, Rifas-Shiman SL, Oken E (2014) Very low maternal lead level in pregnancy and birth outcomes in an eastern Massachusetts population. Ann Epidemiol 24:915–919. 10.1016/j.annepidem.2014.09.007 25444892 10.1016/j.annepidem.2014.09.007 PMC4254591

[CR26] RÍsovÁ V (2019) The pathway of lead through the mother’s body to the child. Interdiscip Toxicol 12:1–6. 10.2478/intox-2019-0001 32189981 10.2478/intox-2019-0001 PMC7061448

[CR27] Ruckart PZ (2021) Update of the blood lead reference value—United States, 2021. MMWR Morb Mortal Wkly Rep. 10.15585/mmwr.mm7043a4 10.15585/mmwr.mm7043a4 PMC8553025 34710078

[CR28] Santana AB, Spelta LEW, Sobalvarro JVM, Podestá MHMC, Garcia RCT, Dos Reis TM, Torres LH (2023) Gestational lead exposure and its effects on fetal/infant development - A systematic review. Reprod Toxicol 117:108342. 10.1016/j.reprotox.2023.108342 36758879 10.1016/j.reprotox.2023.108342

[CR29] Taylor CM, Tilling K, Golding J, Emond AM (2016) Low level lead exposure and pregnancy outcomes in an observational birth cohort study: Dose-response relationships. BMC Res Notes 9:291. 10.1186/s13104-016-2092-5 27260491 10.1186/s13104-016-2092-5 PMC4893212

[CR30] Tembo BD, Sichilongo K, Cernak J (2006) Distribution of copper, lead, cadmium and zinc concentrations in soils around Kabwe town in Zambia. Chemosphere 63:497–501. 10.1016/j.chemosphere.2005.08.002 16337989 10.1016/j.chemosphere.2005.08.002

[CR31] Vigeh M, Saito H, Sawada S (2011) Lead exposure in female workers who are pregnant or of childbearing age. Ind Health 49:255–261. 10.2486/indhealth.ms1192 21173522 10.2486/indhealth.ms1192

[CR32] Vigeh M, Sahebi L, Yokoyama K (2023) Prenatal blood lead levels and birth weight: A meta-analysis study. J Environ Health Sci Eng 21:1–10 37155699 10.1007/s40201-022-00843-w PMC10163201

[CR33] Vigeh M, Yokoyama K, Sahebi L (2025) Prenatal lead exposure and risk of pregnancy-induced hypertension: A systematic review and meta-analysis. BMC Pregnancy Childbirth 25:989. 10.1186/s12884-025-08131-9 41034873 10.1186/s12884-025-08131-9 PMC12487262

[CR34] Wessel L (2025) Prevention of Lead Exposure in the Perinatal Period. J Obstet Gynecol Neonatal Nurs JOGNN S0884–2175(25):00253–00259. 10.1016/j.jogn.2025.08.005 10.1016/j.jogn.2025.08.005 40953824

[CR35] WHO (2024) Guideline on haemoglobin cutoffs to define anaemia in individuals and populations. www.who.int/publications/i/item/9789240088542. Accessed 2 Mar 2026 38530913

[CR36] Yabe J, Nakayama SM, Ikenaka Y, Muzandu K, Choongo K, Mainda G, Kabeta M, Ishizuka M, Umemura T (2013) Metal distribution in tissues of free-range chickens near a lead–zinc mine in Kabwe, Zambia. Environ Toxicol Chem 32:189–192. 10.1002/etc.2029 23059509 10.1002/etc.2029

[CR37] Yabe J, Nakayama SM, Ikenaka Y, Yohannes YB, Bortey-Sam N, Oroszlany B, Muzandu K, Choongo K, Kabalo AN, Ntapisha J (2015) Lead poisoning in children from townships in the vicinity of a lead–zinc mine in Kabwe, Zambia. Chemosphere 119:941–947. 10.1016/j.chemosphere.2014.09.028 25303652 10.1016/j.chemosphere.2014.09.028

[CR38] Yabe J, Nakayama SM, Ikenaka Y, Yohannes YB, Bortey-Sam N, Kabalo AN, Ntapisha J, Mizukawa H, Umemura T, Ishizuka M (2018) Lead and cadmium excretion in feces and urine of children from polluted townships near a lead-zinc mine in Kabwe, Zambia. Chemosphere 202:48–55. 10.1016/j.chemosphere.2018.03.079 29554507 10.1016/j.chemosphere.2018.03.079

[CR39] Yabe J, Nakayama SM, Nakata H, Toyomaki H, Yohannes YB, Muzandu K, Kataba A, Zyambo G, Hiwatari M, Narita D (2020) Current trends of blood lead levels, distribution patterns and exposure variations among household members in Kabwe, Zambia. Chemosphere 243:125412. 10.1016/j.chemosphere.2019.125412 31995873 10.1016/j.chemosphere.2019.125412

[CR40] Yadav G, Chambial S, Agrawal N, Gothwal M, Kathuria P, Singh P, Sharma P, Sharma PP (2020) Blood lead levels in antenatal women and its association with iron deficiency anemia and adverse pregnancy outcomes. J Fam Med Prim Care 9:3106–3111. 10.4103/jfmpc.jfmpc_78_20 10.4103/jfmpc.jfmpc_78_20 PMC7491757 32984181

[CR41] Yamada D, Hiwatari M, Hangoma P, Narita D, Mphuka C, Chitah B, Yabe J, Nakayama SM, Nakata H, Choongo K (2020) Assessing the population-wide exposure to lead pollution in Kabwe, Zambia: an econometric estimation based on survey data. Sci Rep 10:1–11. 10.1038/s41598-020-71998-5 32934309 10.1038/s41598-020-71998-5 PMC7492281

[CR42] Yoshii Y, von Rein I, Munthali K, Mwansa M, Nakata H, Nakayama S, Ishizuka M, Uchida Y (2020) Evaluation of phytoremediation effects of chicken manure, urea and lemongrass on remediating a lead contaminated soil in Kabwe, Zambia. S Afr J Plant Soil 37:351–360. 10.1080/02571862.2020.1772386

[CR43] Zaballa K, Liu A, Peek MJ, Mongelli M, Nanan R (2012) Association between World Health Organization categories of body mass index and relative risks for weight-related pregnancy outcomes: A retrospective cohort study. Obstet Med 5:112–118. 10.1258/om.2012.110091 27582867 10.1258/om.2012.110091 PMC4989705

[CR44] Zhang B, Xia W, Li Y, Bassig BA, Zhou A, Wang Y, Li Z, Yao Y, Hu J, Du X, Zhou Y, Liu J, Xue W, Ma Y, Pan X, Peng Y, Zheng T, Xu S (2015) Prenatal exposure to lead in relation to risk of preterm low birth weight: A matched case-control study in China. Reprod Toxicol 57:190–195. 10.1016/j.reprotox.2015.06.051 26122562 10.1016/j.reprotox.2015.06.051 PMC4843791

